# Inhalation Aromatherapy *via* Brain-Targeted Nasal Delivery: Natural Volatiles or Essential Oils on Mood Disorders

**DOI:** 10.3389/fphar.2022.860043

**Published:** 2022-04-12

**Authors:** Jieqiong Cui, Meng Li, Yuanyuan Wei, Huayan Li, Xiying He, Qi Yang, Zhengkun Li, Jinfeng Duan, Zhao Wu, Qian Chen, Bojun Chen, Gang Li, Xi Ming, Lei Xiong, Dongdong Qin

**Affiliations:** ^1^ School of Basic Medical Sciences, Yunnan University of Chinese Medicine, Kunming, China; ^2^ Department of TCM Pediatrics, Yunnan Provincial Hospital of Traditional Chinese Medicine, Kunming, China

**Keywords:** inhalation aromatherapy, nasal-brain pathway, mood disorders, aromatic herbs, essential oils, anxiety, depression, sleep disorders

## Abstract

Mood disorders, also often referred to as affective disorders, are a group of psychiatric illnesses that severely impact mood and its related functions. The high medical expenditures have placed a significant financial burden on patients and their families. Aromatherapy is an alternative and complementary treatment that utilizes essential oils (EOs) or volatile oils (VOs) to achieve major therapeutic goals. In general, EOs are volatile chemicals that enter the body primarily through skin absorption and/or nasal inhalation. In addition, they can work through oral administration. Inhalation aromatherapy has shown unique advantages for treating mood disorders, especially depression, anxiety and mental disorders such as sleep disorder, which have been validated over the last decade through clinical and animal studies. Accumulating evidence has shown that EOs or VOs can bypass the blood-brain barrier to target brain tissue through the nasal-brain pathway. Subsequently, they act on the cerebral cortex, thalamus, and limbic system in the brain to improve symptoms of anxiety, depression and improve sleep quality. Here, we review the natural aromatic plants’ volatiles or essential oils used commonly as adjuncts to manage mood disorders and illustrate the mechanisms of inhalation aromatherapy, and mainly summarized the application of transnasal inhalation aromatherapy in depression, anxiety, and sleep disorders. We conclude that aromatherapy does not cause side-effects, which is vastly different from commonly used psychotropic drugs. Inhalation aromatherapy *via* brain-targeted nasal delivery offers potentially efficacious treatment for mental disorders and merits further study.

## Introduction

Mood disorders are affective mental disorders characterized by significant and persistent changes in emotion or state of mind (Marvel and Paradiso, 2004; Hasler, 2020). They can originate from psychological disorders, organic damage, nerve injury, side effects of medications used to treat physical or mental disorders, and chronic stress (Jorge and Arciniegas, 2014; Sanacora et al., 2022). The COVID-19 pandemic has severely aggravated the occurrence of mental illness and contributed significantly to the global increase in the prevalence of morbidity and disability (Vigo et al., 2016; COVID-19 Mental Disorders Collaborators, 2021). Studies on the global disease burden have revealed the severity of this ailment (Yun et al., 2016; Feigin et al., 2019). The pathogenesis of which are related to gene-environment interactions. In addition, they are multifactorial illnesses triggered by particular environmental variables in genetically vulnerable people, which can impact their capacity to relate to others and function (Cenit et al., 2017; Briguglio et al., 2020). Severe mood disorders could have serious negative consequences extending to family members (Fekadu et al., 2021). Moreover, the *International Classification of Diseases* (ICD-11) published by the World Health Organization (WHO) reveals that mental disorders include diseases of depression, bipolar illness, anxiety (e.g., panic attacks and phobias), mood-related sleep disorders, and other mood disorders (e.g., compulsive overeating) and conditions associated with post-traumatic stress disorder and more (Krawczyk and Święcicki, 2020). In most cases, mild symptoms can be relieved with a combination of medications and psychotherapy.

It has long been speculated that some essential oils derived from natural aromatic plants can help improve sleep quality and mood disorders by inhalation (Ko et al., 2021). In the early 20th century, aromatherapy was first defined as a medical treatment by a French chemist, *René-Maurice Gattefossé* (Koyama and Heinbockel, 2020). In 1975, *Pierre Franchomme*, a pharmacologist and aromatologist, proposed the concept of the “chemotype,” the true chemical identity of a plant, and listed the critical aromatic compounds that characterize each plant and their influence on its properties—made breakthroughs in aromatherapy. In recent years, use of natural aromatherapy as adjuvant therapy for mental disorders, especially anxiety and depression has increased steadily, and increasing research is being done on the treatment mechanism (Lizarraga-Valderrama, 2021). And furthermore, it has been proven to produce pharmacological effects *via* the use of high-quality essential oils entering the body by the nasal inhalation (through the respiratory system or olfactory nerves), through topical absorption (skin), or through oral administration (digestive system) (Martinec, 2012; Gnatta et al., 2016; Acimovic, 2021). It is important to note that systemic administration of essential oils *via* intraperitoneal injection is often used in animal studies. The results of an animal experiment showed that systemic administration of essential oils induced antioxidant, anti-inflammatory, and γ-aminobutyric acid (GABA) changes to alleviate anxiety-like behavior in rats (Cui et al., 2020). Essential oils used in aromatherapy are hydrophobic liquids containing volatile aromatic molecules extracted in concentrated form from herbs, flowers, and other plant parts (Wang and Heinbockel, 2018). Researchers have analyzed the physiological effects of volatile aromatic molecules from pharmacological and aromatherapy perspectives and suggested that aromatherapy be a natural therapy for patients suffering from anxiety or depression (Tsang and Ho, 2010; Saiyudthong and Marsden, 2011; Amsterdam et al., 2012). Specifically, a significant reason for aromatherapy’s effectiveness in treating mood disorders is the presence of desirable chemical components and biological activities in essential oils such as limonene, linalool, linalyl acetate, geraniol, citronellol, and more. These chemicals have been extensively studied and have shown anxiolytic and antidepressant properties (Setzer, 2009; Vieira et al., 2018; Zhang and Yao, 2019; Agatonovic-Kustrin et al., 2020; Soares et al., 2021).

**FIGURE 1:**
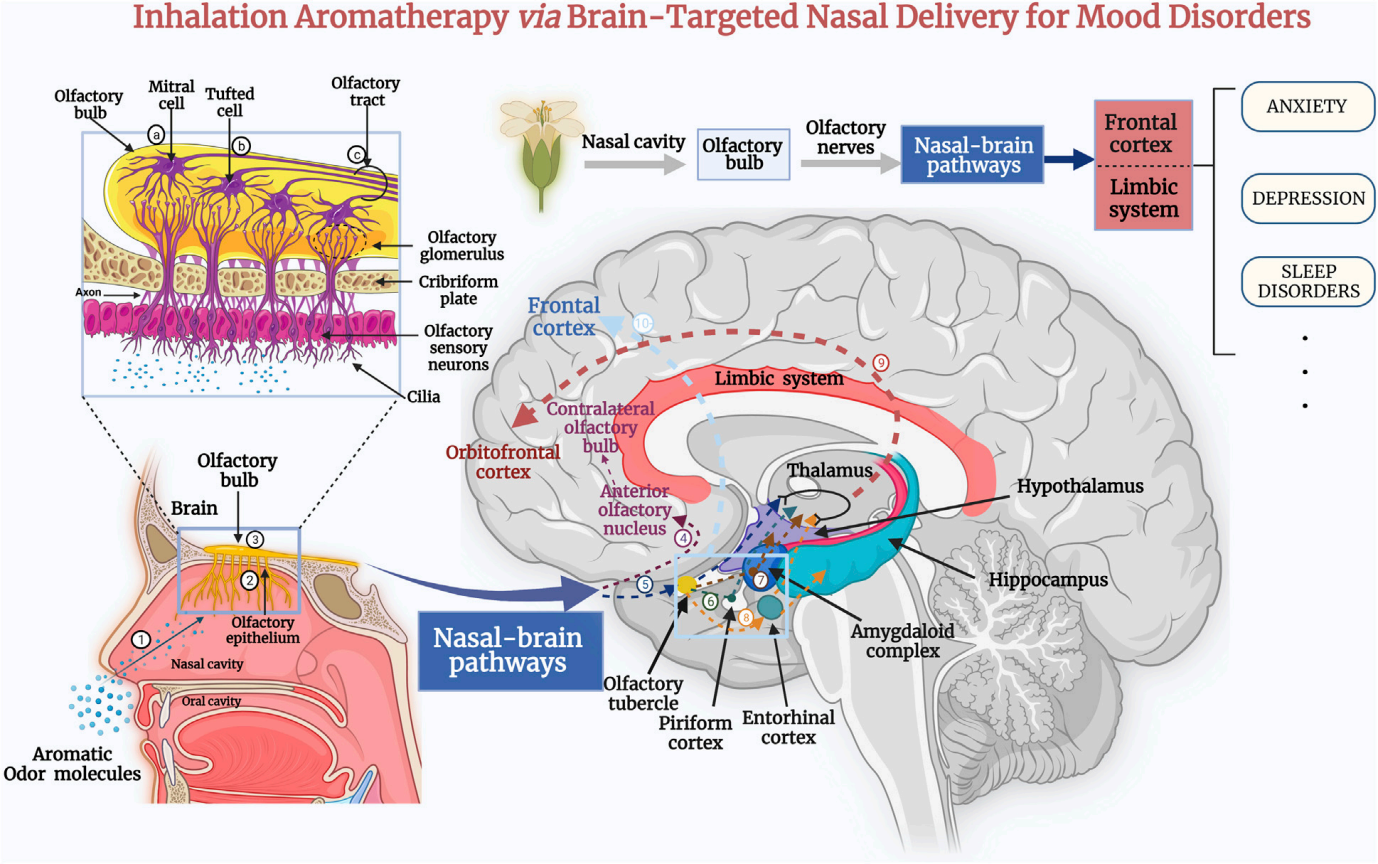
Inhalation of the extracts of aromatic plant *via* the nose sends signals directly to the olfactory system, where odor molecules target therapeutic drugs to brain tissue *via* nasal–brain channels. Subsequently, they act on the cerebral cortex, the thalamus, and the limbic system of the brain, and stimulate the brain to produce neurotransmitters to treat the symptoms of anxiety and depression, as well as improve sleep quality (Lv et al., 2013). The aromatic odor molecules are inhaled through the nasal cavity (1) to reach the olfactory epithelium (2) of the nasal mucosa (Schneider et al., 2018). First-order neurons transmit the odor-evoked response to the olfactory bulb (3). In the olfactory bulb, the axons of mitral cells (a) and some tufted cells (b) (secondary neurons) form the olfactory tract (c). The axons of some mitral cells or lateral branches enter the anterior olfactory nucleus (4) and pass to the contralateral olfactory bulb (Cha et al., 2021). Additional secondary neurons enter the olfactory striatum (medial, lateral, and medial) and then project to central olfactory areas, including the olfactory tubercle (5), piriform cortex (6), amygdala (7), and the entorhinal cortex (8). The entorhinal cortex partially transmits to the hippocampus. Eventually, the central olfactory-area signals are transmitted through the thalamus to the orbitofrontal cortex (9) (Lie et al., 2021). An additional olfactory signaling pathway passes directly from the central olfactory area to the prefrontal cortex (10). These impulses induce the release of neurotransmitters such as serotonin or endorphin, which act as a “bridge” between nerves and other bodily systems (Schneider et al., 2018; Smith and Bhatnagar, 2019).

Inhalation aromatherapy *via* brain-targeted nasal delivery is one of the most common methods of administration in trials of aromatherapy and has evolved from the inhalation of essential oils, in which simple inhalation benefits the emotional wellbeing, tranquility, relaxation, or renewal of the human body (Ali et al., 2015). In clinical applications, transnasal inhalation of essential oils can be used nasal inhaler, vapor diffuser, spraying into the air, vapor balms, or direct inhalation by evaporation using tissue or cotton round (Acimovic, 2021). Notably, inhalation of essential oils or aromatic plant volatile oils can send signals directly to the olfactory system and trigger the brain to produce neurotransmitters e.g., serotonin [5-hydroxytryptamine (5-HT) and dopamine], influence the neuroendocrinological system, neurophysiological brain activity, sympathetic and parasympathetic nervous system, biomarkers changes, psychological and behaviour effects, and to modulate mental disorders further (Masago et al., 2000; Hongratanaworakit, 2004; Lahlou, 2004; Watanuki and Kim, 2005; Strous and Shoenfeld, 2006; Tanida et al., 2008; MPham et al., 2012; Lv et al., 2013; Angelucci et al., 2014). From this, it can be seen that inhalation aromatherapy on mental disorders is due to the pharmacological effect caused by systemic absorption or act on the nervous system, but not only due to the psychological perception of the scent (Scuteri et al., 2019). A potential mechanism for the effects of inhalation aromatherapy on brain function is the activation of nasal olfactory chemoreceptors and subsequent olfactory signaling. Olfaction is not only the oldest and the most vital sense for survival, it is also the only one unaffected by psychological processes (Cook and Lynch, 2008; Schneider et al., 2018; Cha et al., 2021). In humans, ∼300 genes are dedicated to detecting thousands of distinct scent molecules through a vast family of olfactory receptors (Sowndhararajan and Kim, 2016). In recent years, the mechanism of inhaled essential oils delivered to brain targets is being intensively studied. According to neurobiological studies, the olfactory nerve links the olfactory system to the central nervous system, which allows odor information processing. Moreover, higher-order (prefrontal) processes mediate the “smell experience.” If odor molecules contact the nasal mucosa, first-order neurons transmit the odor-evoked response to the olfactory bulb (Schneider et al., 2018). The olfactory tract is a complex system comprising sensory axons and second-order dendrites (mitral and tufted cells) located in the olfactory sulcus of the basal forebrain, and conveys information to several locations within the frontal and dorsomedial lobes (Nagayama et al., 2014; Smith and Bhatnagar, 2019). Olfactory perception starts with the binding of odorant molecules with suitable receptor proteins, and terminates in higher cerebral cortex, making us consciously aware of an odor. Odorous compounds can elicit chemo-electrical transduction pathways to modulate the excitability of the sensory neurons through converting the chemical stimulus into electrical impulses (Buck and Axel, 1991; Breer, 2003). Following that, olfactory sensory neurons convey electrical impulses to the limbic and hypothalamic regions of the brain through the olfactory bulb and upper olfactory cortex. These projections together comprise the primary olfactory cortex. Then, these olfactory areas produce higher-order projections to the orbital prefrontal cortex, amygdala, hypothalamus, basal ganglia, and hippocampus (Figure 1) (Angelucci et al., 2014; Lie et al., 2021). Another potential mechanism is that the essential oil molecules inhaled *via* steam enter the blood circulation by the alveoli of the respiratory system, and subsequently small lipophilic molecules easily cross the blood-brain barrier (BBB) to affect the brain (Faturi et al., 2010; Selvaraj et al., 2017). However, whether this pathway of nasal/respiratory system/circulation system/brain produces pharmacological effects is highly dependent on the drug properties, dose and concentration of the administration (Illum, 2003). In recent years, based on the limitations of dosages and amount of activity in inhalation administration, researchers have focused on the use of nanocarrier technology for transnasal targeting of drugs to the brain to improve drug utilization.

This review summarizes the aromatic oils that may be used to treat mood disorders, assesses the efficacy of inhaled aromatherapy in treating anxiety, depression and sleep disorders (Table 1), and delves into their mechanism of action. In addition, we discuss recent data on the impact of inhalation aromatherapy on the brain and the pathways involved (Figure 1). Further, we summarized animal and clinical data and analyzed transnasal brain-targeted inhaled aromatherapy in anxiety, depression, and sleep disorders. Inhalation aromatherapy holds promise for preventing or treating mood disorders, but additional study is required to grasp the fundamental mechanisms involved. We argue for further clinical and scientific studies on inhalation aromatherapy to treat mental disorders, especially anxiety, depression, sleep disorders.

**TABLE 1:** Use of aromatic plants’ volatiles or essential oils to treat neuropsychiatric disorders.

Scientific name	Main active ingredients	Main indications	Mechanism of action	References
Lavender (*Lavandula angustifolia Mill.*) Essential Oil	Linalyl acetate, linalool, (E)-β- stilbene, limonene	Anxiety Depression Sleep disorder	↑: Parasympathetic nervous system	López et al. (2017); Ozkaraman et al. (2018) ; Mahdavikian et al. (2020); Kim M. et al. (2021)
			↑: Dopamine receptors subtype D_3_	
			↑: Alpha waves in the brain	
			↓: NMDA receptors	
			↓: Serotonin transporter (SERT)	
Roman Chamomile (*Chamaemelum nobile* L.) Essential Oil	Angelic acid, tiglic acid, α-pinene, and 2-methyl butanoic acid	Anxiety Depression Sleep disorder	↑: Mitochondrial function	Kong et al. (2017); Ebrahimi et al. (2021); Jia et al. (2021)
			↑: Expression of parvalbumin mRNA in hippocampus	
			↑: Neuroactive ligand- receptor interactions	
			↑: 5-hydroxytryptamine	
			synapses	
Bergamot (*Citrus bergamia Risso et Poiteau*) Essential Oil	Monoterpenes limonene, monoterpene esters, linalyl acetate, and linalool	Anxiety Depression Sleep disorder	↑: Synaptic transmission	Costa et al. (2010); Donato et al. (2014); Watanabe et al. (2015); Rombolà et al. (2017)
			↑: EEG activity	
			↑: Neuroprotective effects	
Lemon Balm (*Melissa officinalis* L.) Essential Oil	Citral, citronellal, linalool, geraniol and β-caryophyllene-oxide	Anxiety Depression Fatigue	↑: Dopamine	Komiya et al. (2006)
			↑: Serotonin	
Saint John’s wort (*Hypericum perforatum L.*) extracts	Germacrene D, (*E*)-caryophyllene, 2-methyl octane, α-pinene, hypericin, proto-hypericin	Anxiety Depression Sleep disorder	↑: Serotonergic system	Wong et al. (2004); Linde et al. (2008); Di Pierro et al. (2018)
			↓: Monoamine neurotransmitters	
Rhodiola rosea L. (*R. rosea L.*) extracts	Cinnamyl alcohol glycosides such as rosin, rosavin, and the phenylethanoid compound salidroside with its aglycone tyrosol	Anxiety Depression Sleep disorder Fatigue	↑: Neurotransmitters	Panossian et al. (2010); Alperth et al. (2019); Panossian et al. (2021); Kim K. J. et al. (2021)
			↑: BDNF/TrkB signaling pathway	
			↓: Anti-inflammatory action	
			↓: Glucocorticoid receptor	
			↓: Activity of HPA axis	
Cang-ai volatile oil	Eugenol, 1,8-cineole, patchouli alcohol, acetyl eugenol, linalool, linalyl acetate	Depression	↑: Dopamine	Chen et al. (2019)
			↑: 5-hydroxytryptamine	

↑, enhance, activate or increase; ↓, weaken, inactivate or decrease.

## Aromatic Plants and Extracts for Mood Disorders

The essential oils used for aromatherapy have been extracted from aromatic plants and herbs to treat various ailments for centuries. About 17,500 plants have an aromatic scent (Tu et al., 2020). Because of their specific pharmacological functions, essential oils can be used in various ways to stimulate specific physiological responses for symptom relief. For instance, some scents (e.g., *Mentha* × *piperita* L. peppermint oil) can change the endogenous opioid pathways of the brain to alleviate pain and anxiety (Zarzo, 2007; Angelucci et al., 2014). Moreover, aromatic herbal preparations have long been a mainstay for treating anxiety and depression. Volatile oils are active chemicals derived from aromatic herbal remedies that often possess a broad spectrum of biological actions (Wang et al., 2014). Recent research indicates that the chemically active components in essential oils or volatile oils have neuroprotective effects, which may help alleviate depression and anxiety symptoms. Table 1 summarizes the properties of aromatic herbs or essential oils for the treatment of mood disorders.

### Lavender (*Lavandula angustifolia* Mill.) Extract

*Lavandula angustifolia* Mill. is an aromatic plant that belongs to the Labiatae family. It contains high concentration of volatile oils from the aromatic parts of the plant, and is considered one of the most effective over-the-counter aromatic herbal extracts for the treatment of anxiety, depression, and stress (López et al., 2017). Many botanical species of lavender can be used medicinally, and most have a similar chemical composition, including *Lavandula angustifolia* (English lavender), *L. stoechas* (French lavender), *L. latifolia* (a Mediterranean grass-like lavender) (Cavanagh and Wilkinson, 2002; Sanna et al., 2019). The chemical compounds linalyl acetate (3,7-dimethyl-1,6-octadien-3yl acetate), linalool (3,7-dimethylocta-1,6-dien-3-ol), lavandulol, 1,8-cineole, lavandulyl acetate, and camphor are the primary constituents (Da Porto et al., 2009; Carson and Hammer, 2010; Ozkaraman et al., 2018). However, the main chemical composition of lavender oil varies from species to species. A study determined the composition of the essential oils of *Lavandula angustifolia* and *Lavandula latifolia*. Of these, linalool (37–54%), linalyl acetate (21–36%), and (E)-β-stilbene (1–3%) were the most abundant in *Lavandula angustifolia*. In contrast, the higher content of linalool (35–51%), eucalyptol (26–32%), camphor (10–18%), α-pinene (1–2%), α-terpineol (1–2%) and α-bisabolene (1–2%) were found in *L. latifolia* (Carrasco et al., 2015)*.* Linalool and linalyl acetate are the most important constituents of lavender. Linalool contains sedative and narcotic properties, whereas linalyl acetate possesses narcotic properties. The scent of lavender has been studied, and linalool and linalyl acetate have been found to activate the parasympathetic nervous system (Fayazi et al., 2011; Mahdavikian et al., 2020).

An animal study demonstrated that inhalation of lavender oil improves anxiety-like behaviors in rats. After at least 30 min of inhalation, peripheral movements, and defecation in an open field were reduced in anxiety model rats (Shaw et al., 2007). Lavender essential oil improved depression-like behavior, neurogenesis, and synaptic plasticity, and depression-like and anxiety-like behaviors were alleviated significantly by corticosterone administration in animal models. Furthermore, *L. angustifolia* essential oil increases the number of bromodeoxyuridine-positive cells in the rat hippocampus and ameliorates corticosterone-induced neuro-regeneration disorders (Sánchez-Vidaña et al., 2019). Related animal studies have shown that lavender possesses significant receptor-binding affinities and activity on the N-methyl-D-aspartate (NMDA) receptor. Hence, the anxiolytic and antidepressant properties of lavender oil may be due (at least in part) to its regulation of glutamate NMDA receptors and suppression of the serotonin transporter (López et al., 2017).

Research has shown that high-stress levels or continuous exposure to stress can impair human social interaction and lead to social anxiety. A randomized controlled trial showed the effects of lavender oil aromatherapy on anxiety and sleep quality in chemotherapy patients. Lavender oils were administered to the respective intervention groups, and no aromatherapy was administered to the control group. The outcome evaluation indicators were the State-Trait Anxiety Inventory (STAI) and the Pittsburgh Sleep Quality Index (PSQI). This study determined that chemotherapy patients were inhaling three drops of lavender essential oil nightly before sleep can reduce trait anxiety levels and improve sleep quality (Ozkaraman et al., 2018). Kim and others conducted a systematic review and meta-analysis using PRISMA criteria to investigate the efficacy of lavender on anxiety, depression, or physiologic factors in humans. They found that lavender aromatherapy decreased anxiety and despair significantly, and that one administration session strengthened the anxiolytic effects of lavender aromatherapy (Kim M. et al., 2021). However, according to Kang and co-workers, lavender oil is effective in reducing anxiety, but there is some variation in the magnitude of the effect. When analyzing by route of administration, the effect of inhalation *via* intranasal administration is the most prominent (Donelli et al., 2019; Kang et al., 2019). Hence, animal research and clinical trials should be undertaken to gain deeper understanding of the effects of *L. angustifolia* essential oil administered through nasal–brain pathways.

### Roman Chamomile (*Chamaemelum nobile* L.) Essential Oil

Chamomile is an aromatic medicinal plant in the Asteraceae family, widely used by ethnic and traditional medicine, represented by two common varieties *viz*. German Chamomile (*Matricaria chamomilla*) and Roman Chamomile (*Chamaemelum nobile*) (Hansen and Christensen, 2009). Due to its volatile, bioactive phytochemicals, it has been used to treat various diseases. However, there are differences in the main volatile substances of different species of chamomile, an analysis of terpenoid biosynthesis pathways based on co-expression networks showed that the main volatiles of German chamomile are monoterpenes and sesquiterpenes, while the main volatiles of Roman chamomile are esters (Mann and Staba, 1986; Tai et al., 2020). Roman Chamomile essential oils are often used as a mild sedative to calm nerves, decrease anxiety, and cure nightmares, insomnia, and other sleep difficulties (McKay and Blumberg, 2006; Singh et al., 2011). Researchers investigated the effect of inhalation of Roman chamomile essential oil on depressive-like behaviors in Wistar–Kyoto (WKY) rats for 2 weeks. After inhaling Roman chamomile essential oil or one of its main components, α-pinene, depression-like behavior in WKY rats was improved during the forced swimming test (FST). Furthermore, an increase in expression of the proteins involved in oxidative phosphorylation and parvalbumin mRNA expression in the hippocampus were documented. Those findings suggested that mitochondrial function and small parvalbumin-related signaling may be involved in the antidepressant effect of chamomile (Kong et al., 2017). Hashikawa-Hobara and others suggested a new role for Roman chamomile. They found that inhalation of Roman chamomile essential oil combined with chlorpromazine reduced drug-resistant depression-like behavior in mice, and had a crucial role in drug-resistant depression-like behavior. Their results may help people suffering from drug-resistant depression and provide a target for innovative antidepressant therapies (Hashikawa-Hobara et al., 2019). Future emphasis should be placed on animal studies with chamomile involving animal models of various psychiatric disorders, which will help develop chamomile as a promising therapeutic agent (Srivastava et al., 2010).

*Matricaria chamomilla* L*.* has multi-target and multi-pathway characteristics to treat anxiety disorders and depression. Jia and others undertook research based on network pharmacology and database mining. They revealed that the active components of Roman chamomile participate in neuroactive ligand-receptor interactions, 5-HT release into synapses, the cyclic adenosine monophosphate signaling pathway, and neurotransmitter-binding pathway, and that LRRK2 may be a critical gene in Roman chamomile for the treatment of anxiety disorders (Jia et al., 2021). In a recent three-arm parallel randomized controlled trial, 183 participants were included in the study and randomized into three groups (*n* = 61): lavender, chamomile, and control groups. Participants in the experimental group inhaled three drops of 1.5% lavender and chamomile essential oils for 30 nights. Participants in the control group inhaled only distilled water similarly. Compared to the control group, the lavender and chamomile groups showed statistically significant improvements in depression, anxiety, and stress levels immediately and 1 month after the intervention (*p* < 0.01). The trial found that inhalation aromatherapy with chamomile essential oils and lavender extract reduced depression, anxiety, and stress levels in older community-dwelling people (Ebrahimi et al., 2021). Effectiveness of using chamomile essential oil on stress symptoms and stress management in clinical practice is required. Further studies on the effectiveness of chamomile essential oil in clinical applications for psychiatric disorders are needed, and further search is more focused on antidepressant and anxiolytic pharmacological mechanisms.

### Bergamot (Citrus *bergamia* Risso et Poiteau) Extracts

*Citrus bergamia* Risso et Poiteau is a species of plant in the Rutaceae family (subfamily Esperidea), also known as “Bergamot.” Bergamot essential oil (BEO) is a volatile oil preparation obtained by rasping and cold pressing the peel of the fruit (Mannucci et al., 2017). The main active ingredients of BEO are composed of 93–96% volatile and 4–7% non-volatile components. The volatile components mainly include monoterpene limonene accounts for 25–53% and a large number of oxygenated compounds, such as linalool, linalyl acetate, γ-terpinene, and β-pinene (Mondello et al., 1998; Moufida and Marzouk, 2003; Costa et al., 2010; Donato et al., 2014; Navarra et al., 2015). In addition, linalyl acetate is also a highly represented monoterpene in bergamot oil, sometimes almost as abundant as limonene (Poiana et al., 2003; Verzera et al., 2003; González-Mas et al., 2019). Its non-volatile component 5-MOP can cause phototoxicity, however, modern vacuum distillation of bergamot peel technology can obtain high-quality *C. bergamia* essential oil utterly free of 5-MOP, and chemical properties are comparable to those of cold-pressed oil (Belsito et al., 2007). Animal model studies have shown that the main components of bergamot can affect the synaptic transmission, regulate electroencephalography (EEG) activity, and have neuroprotective effects (Rombolà et al., 2009; Bagetta et al., 2010). One study in rats focused on the anxiolytic and sedative effects of bergamot essential oils. The behavioral effects were compared with benzodiazepine diazepam by subjecting rats to the forced swimming test (FST), open field test (OFT), and elevated maze test (EMT). The results indicated that bergamot alleviated anxiety-like behavior in rats, thereby adding to the understanding of the pharmacological profile of bergamot and bolstering its rational use in aromatherapy (Rombolà et al., 2017).

In 2015, Watanabe and others undertook a random crossover trial examining the effects of inhalation of the vapor of bergamot essential oil for 15 min on 41 healthy women. They measured the salivary level of cortisol and heart rate, as well as self-reported anxiety, fatigue, and emotional state. Volatile oils were inhaled into the lungs *via* the nose and transported into the bloodstream *via* the alveoli, and elicited significant psychoactive and physiological effects. The results showed that inhalation of bergamot oil helped slow-down anxiety-induced tachycardia, reduce salivary cortisol levels, and improve negative mood and fatigue scores significantly (Watanabe et al., 2015). In 2011, Hongratanaworakit showed that bergamot essential oil helped treat depression. The analyses were based on the blood pressure, pulse rate, respiration rate, and skin temperature. Compared with placebo, bergamot oil reduced the pulse rate and blood pressure significantly (Hongratanaworakit, 2011). The studies cited above provide evidence for clinical use of the volatile oils of *C. bergamia* essential oil for treating depression or anxiety, but additional high-quality evidence is required to support such use. Rigorous animal studies and high-quality clinical trials on treating mental disorders with Bergamot essential oil are highly needed, and this is a worthwhile direction for the future.

### Essential Oil from *Melissa officinalis* L

Lemon Balm (*Melissa officinalis* L.) is an aromatic medicinal herbal plant from the Labiatae family (Mint family). The *Melissa officinalis* L. essential oils are widely used in traditional medicine to treat many mental disorders such as depression, anxiety, insomnia, anxiety-induced heart palpitations and stress (Shakeri et al., 2016). Not only does it boost mood, but it also helps alleviate depression-related symptoms such as “brain fog,” but the mechanism of action is incompletely understood. Although over 100 chemicals have been identified in *M. officinalis*, the main components of the essential oil are citral, citronellal, linalool, geraniol, and β-caryophyllene-oxide (Miraj et al., 2017). A systematic review and meta-analysis evaluated the effects of *Melissa officinalis* L. essential oil as an herbal remedy on anxiety and depression and its side effects in clinical trials. The abstracts of 68 clinical research studies and the full text of 27 articles were analyzed, 17 studies were excluded after evaluation because they were not randomized controlled trials or did not have a control group. Finally, only 10 articles were included in the qualitative synthesis and six in the quantitative synthesis (meta-analysis). According to the results of the meta-analysis, lemon balm essential oil significantly improved the symptoms of anxiety (standardized mean difference, SMD: 0.98; 95% CI: 1.63 to 0.33; *p* = 0.003) and depression (SMD: 0.47; 95% CI: 0.73 to 0.21; *p* = 0.0005), without serious side effects compared to placebo (Ghazizadeh et al., 2021). Although some animal and clinical studies about inhalation of lemon balm essential oil have been undertaken in recent years, limitations exist due to the high heterogeneity among clinical studies, the small sample size of clinical trials, differences in statistical methods, and the lack of in-depth research on pharmacological effects and mechanisms. In the future, further high-quality randomized controlled trials are needed to clarify the clinical efficacy of *Melissa officinalis* L. oils, and research on the mechanism of action and efficacy also should be increased.

### Saint John’s Wort (*Hypericum perforatum* L*.*) Extracts

Saint John’s wort (SJW), also known as *Hypericum perforatum* L*.,* the extracts can inhibit reuptake of monoamine neurotransmitters, and is often used as an antidepressant. The main active ingredients of *Hypericum perforatum* L*.* extracts are hypericin, proto-hypericin, pseudohy-pericin, proto-pseudohypericin. Its primary use is as an over-the-counter anti-depressive or anxiolytic (Hamid et al., 2017; Zirak et al., 2019). Numerous research has shown that a multi-fractionated SJW extracts improve therapeutic results in patients suffering from depression. Nevertheless, the exact mechanism of action of SJW (and most of the compounds involved) is not known (Di Pierro et al., 2018).

Yu and others discovered that the levels of 5-hydroxy indole acetic acid (5-HIAA) in the cerebral cortex, hypothalamus, hippocampus, and caudate nucleus of mice increased dramatically 3 h after treatment with SJW extracts at concentrations as low as 10 mg/kg body weight. The effects of SJW extract are compatible with the involvement of the serotonergic system (Yu, 2000). Yu and others studied the effects of SJW extracts and the influence of the tricyclic antidepressant (TCA) imipramine on the transcription of hypothalamic genes in rats. They discovered significant correlations between six genes that were regulated directly. Research into the mechanism of action of the purported therapeutic effect of SJW could reveal new processes, novel chemicals, and new biological targets for the development of antidepressant drugs (Wong et al., 2004). Linde and others examined 29 trials involving 5,489 individuals who had depression, and compared therapy with SJW extracts for 4–12 weeks with placebo treatment or conventional antidepressants. The SJW extracts outperformed a placebo in terms of efficacy and had fewer adverse effects than typical antidepressants. Furthermore, it was better tolerated than prescription medications (Linde et al., 2005; Linde et al., 2008). Therefore, the pharmacological effects and mechanisms of SJW extracts in the treatment of mood disorders need to be further investigated in the future.

### *Rhodiola Rosea* L*. (R. Rosea* L*.) Extracts*

*R. Rosea* L*.* is considered a “universal” aromatic botanical, and extracts can be employed to treat fatigue, depression, cognitive dysfunction, and nerve disorders (Brinckmann et al., 2021). The main bioactive compounds of *Rhodiola Rosea* L*.* are phenylpropanoids such as cinnamyl alcohol glycosides rosin and rosavin. Furthermore, the phenylethanoid molecule salidroside contains the aglycone tyrosol (Ma et al., 2018; Alperth et al., 2019). Phenylpropane derivatives mediate the adaptogenic action of R. Rosea preparations (e.g., rosavin) and phenylethylene derivatives (e.g., tyrosol and salidroside), which have pleiotropic pharmacological effects on the neuroendocrine and immune systems (Panossian et al., 2021). The rhizome of *R. Rosea* L. (and the chemicals isolated from it) have been shown to protect neuronal PC-12 cells from oxidative stress and demonstrate mild acetylcholinesterase inhibition, respectively (Kim K. J. et al., 2021). Several clinical studies have demonstrated that using the *R. Rosea* extract SHR-5 regularly has an anti-fatigue effect and promotes cognitive function while decreasing “burnout” in individuals with chronic fatigue syndrome. Moreover, *R. Rosea* has shown promising benefits in treating mild-to-severe depression and generalized anxiety disorder (GAD) (Panossian et al., 2010). One randomized phase-III pilot trial was conducted to determine the efficacy and safety of a standardized extract SHR-5 of the rhizomes of *R. Rosea* L. in individuals experiencing a bout of mild-to-moderate depression. When the extract was taken at 340 mg every day for 6 weeks, a significant decrease in the total level of symptoms of depression (e.g., sleeplessness, mood instability, and somatization) was noted. At greater doses (four pills a day for 6 weeks), a considerable increase in self-esteem was observed (Darbinyan et al., 2007). Future studies may focus on the pharmacological mechanism of *R. Rosea* on mild-to-moderate depression.

### Cang-ai Volatile Oil

Cang-ai Volatile oil (CAVO) is an inhalational preparation employed to treat depressive and emotional disorders, and extracted from the ethnic aromatic herbs such as *Cyperus rotundus* L, Mugwort (*Artemisia vulgaris* L.), patchouli (*Pogostemon cablin (Blanco) Benth.*), clove (*Syzygium aromaticum* (L*.*) *Merr. & L.M.Perry*) and Perrin (*Pimpinella anisum* L.)*.* The top-10 volatile compounds in CAVO identified by gas chromatography-mass spectrometry (GC-MS) are eugenol, 1,8-cineole, patchouli alcohol, acetyl eugenol, linalool, linalyl acetate, caryophyllene, terpinene-4-ol, cineol, and terpineol. CAVO has been shown to ameliorate depression-like behavior in animal studies, and be better than the traditional oral route of antidepressants, the mechanism of action appears to be related to dopamine and 5-HT (Chen et al., 2019). This observation suggests a new aromatic volatile oil preparation for anti-depression medications. In this way, more meaningful pharmacological evidence for efficacious and safe treatments can be obtained. In addition, preliminary results for self-reported pre-and post-tests in patients with depressive tendencies on CAVO inhalation have been obtained. In the future, more clinical trials will be conducted to observe the safety and efficacy of CAVO.

## Aromatherapy Inhalation for Anxiety

Anxiety disorders are the most frequent mental ailment, with a global prevalence ranging between 2.4 and 20% per nation. According to the WHO, 3.6% of the world’s population—around 264 million people—suffer from anxiety (Baxter et al., 2013; Bandelow and Michaelis, 2015; Munir and Takov, 2021). It is a type of dread that emerges in response to potentially dangerous or stressful events, and results from a complex interaction of biological factors, as well as psychological, temperamental, and environmental elements (Thibaut, 2017).

Anxiety disorders include (but are not limited to) panic disorder/agoraphobia (PDA), GAD, social anxiety disorder (SAD), and obsessive-compulsive disorder (OCD) (Giacobbe and Flint, 2018). Panic disorder (with or without agoraphobia) is the most common, accounting for 6.0% of all types, followed by social phobia (2.7%) and GAD as the most common phobias (2.2%) (Craske et al., 2017). Each subcategory of anxiety disorders has its own set of symptoms and diagnostic criteria, but ICD-11 identifies the common symptoms apprehension, motor overactivity, and autonomic overactivity (Reed et al., 2019). Several biological anomalies have been implicated in the pathophysiology of anxiety disorders. The gamma-aminobutyric acid (GABA), norepinephrine, and 5-HT systems have critical roles in modulating the emotional circuitry that underlies anxiety and depression, which are closely connected (Romana et al., 2020).

For anxiety disorders, various treatment options, such as medication and psychotherapy, are available. These treatments may exert their advantages by top-down or bottom-up regulation of abnormal brain activity, respectively (Bandelow et al., 2017; Giacobbe and Flint, 2018). Mild anxiety disorder, in general, does not require excessive treatment, but treatment is indicated if patients manifest significant discomfort or experience disorder-related consequences. For example, subsequent depression, suicidal thoughts, or alcohol misuse are possible outcomes (Bandelow et al., 2017). First-line therapies for anxiety include lifestyle modifications, cognitive behavioral therapy, selective serotonin reuptake inhibitors (SSRIs), or serotonin-norepinephrine reuptake inhibitors (SNRIs) (Bandelow et al., 2015; Mangolini et al., 2019; Garakani et al., 2020). Since the 1980s, researchers have conducted upwards of 10,000 animal studies of nearly 1,500 medications for anxiety, and the number of such studies has shown a marked increase in recent years. However, many studies have not yielded satisfactory results. In many cases, severe adverse drug reactions, dependence, and poor treatment outcomes have persisted (Griebel and Holmes, 2013). There is an immediate need for safe and efficacious treatments and medications for anxiety. The number of studies on inhalation aromatherapy for anxiety disorders has been increasing recently. In general, chamomile, lavender, bergamot, clary sage, rosemary, ylang-ylang, frankincense, and damask rose are used commonly as “anti-anxiety oils.” Essential oils to treat anxiety have been used throughout Europe for many years (Seol et al., 2010). Also, the chemical and biological properties of essential oils have resulted in the development of crucial treatment strategies for anxiety disorders (Fernandes et al., 2021). Essential oils have fewer side effects and more administration methods than traditional anti-anxiety medications. Among them, inhalation has been the most common method of administration in trials of aromatherapy, and the most efficacious (Zhang and Yao, 2019).

Among anxiety-related research items, the lavender essential oil has received the most attention. Franco and others evaluated the reduced anxiety effect of lavender aroma on women before breast surgery. Inhalation of lavender-fragrance aromatherapy treatments reduced anxiety before surgery (Franco et al., 2016). A randomized, double-blind, placebo-controlled clinical study by Farshbaf-Khalili and others focused on anxiety in postmenopausal women. They compared the effects of inhaling oils of lavender or bitter-orange, and found them to have a beneficial impact on anxiety in this population (Farshbaf-Khalili et al., 2018). A randomized controlled study investigating the effect of lavender-oil inhalation on vital signs and anxiety revealed that inhalation aromatherapy was favorable for anxious individuals about to undergo surgical procedures under local anesthesia (Karan, 2019). Guo and others analyzed the efficacy of inhalation aromatherapy on preoperative anxiety. They discovered evidence to support using aromatherapy to alleviate preoperative anxiety in adults. Their results suggested that aromatherapy inhalation was the most practical and feasible mode of administration, and had the advantage of a short duration of administration (20 min for each session). Hence, recent research suggests that lavender oil, preparations of citrus species, and rose oil are the most common and efficacious fragrance preparations for anxiety disorders (Guo et al., 2020). With regard to using essential oils to treat anxiety, initially researchers examined the direct effects of scent on the brain through EEG and functional imaging. They found that the essential oils of rose, lavender, lemon, and peppermint had anti-anxiety effects, and more in-depth research has been conducted in recent years (Lehrner et al., 2005; Bradley et al., 2007; Zhang et al., 2013). Inhaled aromatherapy could reduce preoperative anxiety, but data from primary studies are needed to improve evidence quality (Men et al., 2021).

Animal experiments have shown that inhalation of essential oils can prevent or relieve anxiety symptoms. These beneficial effects may result from modulation of monoamine levels, induction of neurotrophic factors expression, regulation of the endocrine system, and promotion of neurogenesis (Fung et al., 2021). However, an animal experiment has shown that olfactory deficit induced by zinc (zinc gluconate + zinc acetate) did not impair the anxiolytic effects of lavender essential oil inhalation in the marble-burying test. This study demonstrated that the active compounds of lavender oil might enter the systemic circulation and central nervous system through the respiratory system in the absence of olfaction, ultimately activating the relevant receptors to improve anxiety symptoms (Chioca et al., 2013). In future studies, researchers can evaluate the importance of the olfactory system through pharmacological and physiological alterations induced by inhaled essential oils in olfactory impairment animal models. In another animal study, the authors reported that the sedative effect of inhaling a lavender-Roman chamomile oil mixture was impaired by reduced olfactory function (Kagawa et al., 2003). It can be concluded that the olfactory system has an essential role in inhalation aromatherapy, and olfactory impairment on the effects of inhalation is also related to the different types and active ingredients of the essential oils. We can synthesize that the mechanism of transnasal inhalation of essential oils in psychiatric disorders is a function of multiple factors. Researchers should undertake more rigorous animal studies to gain more insight into the mechanisms of inhalation aromatherapy.

## Aromatherapy Inhalation for Depression

Depression is a prevalent mental illness and “mood illness” that manifests *via* a mix of emotional (sadness and anhedonia), cognitive (thinking problems and inability to focus), and somatic symptoms (changes in appetite and insomnia). Approximately 280 million people worldwide suffer from depression. The incidence of depression varies significantly according to geographic location, and depression may increase dramatically in the next decade (Wang, 2021). It is characterized by the profound emotions of melancholy, hopelessness, despair, and an inability to find pleasure in routine activities, as well as changes in sleep and food habits, fatigue, and suicidal thoughts (Sánchez-Vidaña et al., 2019).

Mainstream interventions for depression have relied primarily on medication, such as antidepressants, which may have unacceptable side effects, such as headache, insomnia, nausea, potential drug interactions, or the danger of overdose (Bauer, 2007). Antidepressant medicines may be used as first-line therapy for depressive disorder and are classified into several types. The antidepressants used most often are SSRIs, SNRIs, TCAs, and monoamine oxidase inhibitors (Ramachandraih et al., 2011; Hillhouse and Porter, 2015; Kok and Reynolds, 2017; Zanos and Gould, 2018; InformedHealth.org, 2021). The American College of Physicians advises that practitioners treat individuals with major depressive disorder using cognitive behavioral therapy or second-generation antidepressants (Qaseem et al., 2016). However, ∼30% of persons who use SSRIs for depression experience no response. In addition, the first-line drugs used to treat depression (SSRIs) often have severe side-effects, which stops patients taking them or to lose confidence in treatment. As a result, many people suffering from depression do not achieve remission of symptoms and have to endure relapses and more functional impairment (Adell et al., 2005; Arroll et al., 2005). This phenomenon has pushed patients and researchers to seek more efficacious alternative medicines, particularly in the early phase of treatment. Due to the limitations of those traditional methods and antidepressants, there is a growing interest in using aromatic naturopathy as an alternative therapy. As a complementary approach, inhalation aromatherapy is used widely for treating depression (Koo, 2017; Liang et al., 2021). Numerous studies have indicated that some of the critical constituents of essential oils may reduce depressive symptoms markedly *via* nasal–brain pathways, including those in patients with severe depressive disorder, postpartum women, postmenopausal women, and cancer patients (Chan et al., 2015; Han et al., 2017). In addition, the researchers discovered that citrus scents containing 95% citral were often more appealing and pleasant to people who felt sad (Pause et al., 2001). Inhalation of aromatic molecules through the nose is mild and has few adverse effects, which makes it an attractive option for treating depression (Yim et al., 2009; Lv et al., 2013). And they work more quickly than other methods because the blood-brain barrier does not interfere with the effects. In this way, aromatherapy looks to be a straightforward, cost-effective, and low-risk adjuvant approach for treating depression (Sánchez-Vidaña et al., 2017).

The use of animal models has enabled elucidation of different molecular pathways for treating depression by essential oils, such as the hypothalamic-pituitary-adrenal axis, sympathetic nervous system, cyclic adenosine monophosphate response element-binding protein signaling pathway, and neurotransmitter systems (e.g., serotonergic, dopaminergic, and GABAergic pathways) (Guillemain et al., 1989; Tafet et al., 2001; Raison et al., 2006; Chung et al., 2011; Slavich and Irwin, 2014; Chen et al., 2016; Xiong et al., 2018). de Sousa and others used a rodent model of depression and demonstrated the antidepressant activity of the essential oils of several plants: *Acorus tatarinowii* Schott, *Asarum heterotropoides* F. Schmidt, *Litsea glaucescens* Kunth, *Mentha × Piperita* L, *Citrus limon* (L.) Osbeck, *Eugenia uniflora* L, *Lavandula angustifolia* Mill, *Perilla frutescens* (L.) Britton, *Salvia sclarea* L, *Rosmarinus officinalis* L, *Schinus terebinthifolius* Raddi, and *Syzygium aromaticum* (L.) (de Sousa et al., 2017). A behavioral experiment in a rat model of depression using the FST examined the antidepressant effects of the essential oils of chamomile (*A. nobilis*), clary (*Salvia sclarea*), rosemary (*Rosmarinus officinalis*), and lavender (*L. angustifolia*) in rats. They showed that these oils improved depressive-like behavioral effectively (Seol et al., 2010). In addition, Bagci and others investigated the positive effects of the scopolamine component of *Anthriscus nemorosa* essential oil on memory, anxiety, and depression in rats: their behavior improved (Bagci et al., 2016). Notably, one animal study revealed that lemon oil increased the metabolic turnover of 5-HT markedly in the prefrontal cortex and striatum to enhance 5-HT function, which demonstrated a mechanism of action comparable with that of SSRIs (Komiya et al., 2006; Cowen, 2008). Although various animal studies on the treatment of depression with inhaled essential oils, more sophisticated experiments should be designed to investigate the pharmacological effects.

## Aromatherapy Inhalation for Sleep Disorders

Sleep is vital for sustaining physiological and psychological welfare (Karadag et al., 2017). Numerous mental disorders and sleep disorders show a bidirectional relationship. Patients suffering from anxiety, depression, and other mood disorders have persistent difficulties obtaining a decent night’s sleep. Sleep disturbances can contribute to the risk of mental-health illnesses (Santamaria and Iranzo, 2014; Freeman et al., 2020; Momen et al., 2020). In addition, many antipsychotic agents affect sleep and sleep architecture. Even though sedative-hypnotics may enhance sleep quality significantly, these medications have adverse effects, are addictive, and do not lead to sufficient sleep (Pagel and Parnes, 2001; Hajibagheri et al., 2014). Hence, the application of a less harmful relief method with fewer adverse effects is significant (Hajibagheri et al., 2014; Jodaki et al., 2021).

From ancient times, aromatherapy has been an effective natural therapy for sleep problems, through massage and inhalation using essential oils are the main strategies (Bikmoradi et al., 2015; Guadagna et al., 2020; Mahdavikian et al., 2020). Numerous essential oils have been used to treat sleep difficulties, including lavender oils and peppermint oils (Blackburn et al., 2017; Salamati et al., 2017; Mahdavikian et al., 2020; Malloggi et al., 2021). Research has shown that bergamot oil can reduce blood pressure and the heart rate and aid sleep. Moreover, jasmine and frankincense have been shown to aid restless sleep and improve sleep quality. Takeda and co-workers explored the benefits of inhalation aromatherapy on sleep disturbances in elderly dementia patients by placing essential oils on towels wrapped around their pillows each night. They measured sleep latency, total sleep time, sleep effectiveness, duration of the most extended sustained sleep phase, waking time after sleep onset, early-morning awakening, total daytime sleep, and assessed the Neuropsychiatric Inventory. Inhalation aromatherapy had a beneficial impact on sleep-disruption symptoms in that population (Takeda et al., 2017). Moreover, aromatherapy using the oils of sweet-orange and lavender have been suggested to improve sleep quality and reduce tiredness in hemodialysis patients (Muz and Taşcı, 2017). Another comprehensive study and meta-analysis determined that aromatherapy significantly improved sleep quality and was quick-acting, simple to apply, and did not require additional equipment (Hwang and Shin, 2015). Recently, Zhong and others investigated the compatibility of using the essential oils of Compound Anshen with those of lavender, sweet orange, and sandalwood with sedative and hypnotic properties. They demonstrated improved sleep quality by combining essential oils and blends. Overall, aromatherapy has improved sleep quality in healthy and unwell people, especially if used as inhalation rather than massage treatment (Hwang and Shin, 2015; Zhong et al., 2019). The molecules in essential oils entering the limbic system of the brain through the nasal passages simultaneously affect GABA receptors in the hypothalamus, which are crucial for maintaining sleep. Hence, aromatherapy is quite popular and used commonly to manage sleep quality (Tang et al., 2021).

## Conclusion and Perspectives

In summary, inhalation aromatherapy *via* brain-targeted nasal delivery can be an effective option for improving depression, anxiety, and sleep disorders. When used in inhalation aromatherapy, the advantages of essential oils are high permeability, fast metabolism, non-retention, and low toxicity. Since essential oils are bioactive molecules and have a molecular weight of less than 300 Da (Dhifi et al., 2016), considered safe and biocompatible with a great range of therapeutic applications due to their heterogeneous composition of fatty acids, terpenes, triterpenes, and many other lipophilic components. In addition, a few minutes of inhalation of essential oils *via* the nose can affect the limbic system, and all leave the body by urination, excretion, breathing, and pores in 4–20 h after use (Barradas and de Holanda e Silva, 2021). Notably, the active chemical components of the essential oils or volatile oils used in aromatherapy have fewer side effects than traditional medications for treating mental disorders, but the safety and purity of oils must be considered. Under the supervision of a physician, essential oils of assured quality and demonstrated efficacy should be selected to improve depression or anxiety symptoms. Although the use of volatile essential oils for transnasal administration is common in aromatherapy, inhalation of concentrated forms of essential oils may pose a risk of eye and skin irritation, so direct inhalation of pure essential oils is not recommended. There is an urgent need to clarify the safe dose of inhaled essential oils in clinical applications. One research described a series of methods to assess the efficacy of inhaled essential oils, and clarified that EOs intake dose-relationships with the efficacy of brain function. In general, the inhalation of EOs indicated a dose-dependent relationship with efficacy (Aponso et al., 2020).

Taken together, those data suggest that inhalation aromatherapy may have a more excellent therapeutic range than thought previously, especially in the domain of mental diseases (Perry and Perry, 2006; Ayaz et al., 2017). In the future, more universities, research centers, and medical institutions should conduct qualitative and quantitative analyses of aromatic drugs and extract their components. Also, more multicenter, large-sample, high-quality randomized controlled trials on inhaled aromatherapy for mood disorders are needed. In particular, the use of doses and treatment protocols need to be optimized. We hope that transnasal aromatherapy will lead to further breakthroughs on research into mood disorders.
